# Long noncoding RNA PCAT1, a novel serum-based biomarker, enhances cell growth by sponging miR-326 in oesophageal squamous cell carcinoma

**DOI:** 10.1038/s41419-019-1745-4

**Published:** 2019-07-04

**Authors:** Lijie Huang, Yan Wang, Jiao Chen, Yu Wang, Yabing Zhao, Yali Wang, Yunping Ma, Xin Chen, Wenzhong Liu, Zhengzheng Li, Lianmei Zhao, Baoen Shan, Xin Dong, Dan Li, Shujuan Shao, Yongmei Song, Qimin Zhan, Xuefeng Liu

**Affiliations:** 1 Institute of Cancer Stem Cell, Dalian Medical University, 116044 Dalian, China; 2 State Key Laboratory of Molecular Oncology, National Cancer Center/National Clinical Research Center for Cancer/Cancer Hospital, Chinese Academy of Medical Sciences and Peking Union Medical College, 100021 Beijing, China; 3 Key Laboratory of Carcinogenesis and Translational Research (Ministry of Education/Beijing), Laboratory of Molecular Oncology, Peking University Cancer Hospital & Institute, 100142 Beijing, China; 4 Shenzhen Peking University-the Hong Kong University of Science and Technology (PKU-HKUST) Medical Center, Peking University Shenzhen Hospital, 518035 Shenzhen, China; 5 Key Laboratory of Proteomics, Dalian Medical University, 116044 Dalian, China; 6 Department of Neurosurgery, Beijing Tiantan Hospital, Capital Medical University, 100050 Beijing, China; 7 Research Center, The Fourth Hospital of Hebei Medical University, 050011 Shijiazhuang, China; 8 Department of Clinical Laboratory, National Cancer Center/National Clinical Research Center for Cancer/Cancer Hospital, Chinese Academy of Medical Sciences and Peking Union Medical College, 100021 Beijing, China

**Keywords:** Tumour biomarkers, Cell growth

## Abstract

Long noncoding RNAs (lncRNAs) play important roles in the development and progression of human cancers. The lncRNA prostate cancer-associated transcript 1 (PCAT1) has been reported to be involved in multiple human cancers, including oesophageal squamous cell carcinoma (ESCC). However, the detailed biological functions, underlying mechanisms and clinical relevance of PCAT1 in ESCC remain unclear. Here, we confirmed that PCAT1 was highly expressed in ESCC tissues and cell lines. Knockdown of PCAT1 inhibited the growth of ESCC cells, whereas overexpression of PCAT1 showed the opposite effect both in vitro and in vivo. Moreover, knockdown of PCAT1 arrested the cell cycle at G2/M phase, reduced the expression of cyclin B1 and CDC2, and caused cells to be more sensitive to paclitaxel. Furthermore, PCAT1 could bind to miR-326, a tumour suppressor in diverse human cancers. Rescue experiments revealed that enforced expression of miR-326 attenuated the promotive effect of PCAT1 on ESCC cell growth. In addition, we discovered that PCAT1 was present in ESCC cell-derived exosomes, was higher in the serum of ESCC patients than those of healthy volunteer donors, and promoted cell growth through exosomes. Thus, our data indicate that PCAT1 promotes ESCC cell proliferation by sponging miR-326 and may serve as a non-invasive biomarker for ESCC.

## Introduction

Oesophageal cancer is considered one of the most commonly diagnosed and lethal cancers worldwide^1,2^. The 5-year relative survival rate of patients with oesophageal cancer is very poor, which is associated with its diagnosis at advanced stages^3^. There are two major histological types of oesophageal cancer: oesophageal adenocarcinoma (EAC) and oesophageal squamous cell carcinoma (ESCC). In China, approximately 90% of oesophageal cancers are ESCC, and China has the largest number of ESCC cases worldwide^4^. Thus, a better understanding of the molecular mechanisms driving ESCC progression will facilitate the identification of novel diagnostic markers and therapeutic targets to improve ESCC prognosis.

Long noncoding RNAs (lncRNAs) are a class of RNA transcripts longer than 200 nt that are often polyadenylated and spliced and lack protein-coding potential^5^. LncRNAs have been functionally characterized to carry out regulatory roles in diverse biological processes, such as cell cycle regulation^6^, lipid metabolism^7^, cell differentiation^8^ and innate immune response^9^. Furthermore, increasing evidence indicates that lncRNAs are dysregulated in many human cancers and are involved in carcinogenesis as tumour suppressors or oncogenes, which may provide new opportunities for cancer diagnosis and treatment^10–12^.

Exosomes are membrane vesicles containing a wide variety of molecules, such as non-coding RNAs (ncRNAs) and proteins, and can be secreted by most mammalian cell types, including cancer cells^13^. Exosomes are now recognized as critical messengers of intercellular crosstalk by transferring molecular cargo to recipient cells and have potential clinical applications in cancer diagnosis and/or therapy^14–16^. In particular, cancer-derived exosomal ncRNAs play a key role in cell-cell communication to promote tumour progression and exist in body fluids, where they can serve as non-invasive biomarkers^17–19^. For example, lncARSR, an lncRNA highly expressed in sunitinib-resistant renal cell carcinoma (RCC) cells, can be packaged into exosomes and transmitted to sunitinib-sensitive RCC cells, leading to the dissemination of sunitinib resistance^20^. Moreover, the lncARSR levels are higher in the serum of RCC patients than healthy donors and predict sunitinib response in RCC patients^20^.

Prostate cancer-associated transcript 1 (PCAT1) was originally identified as a prostate cancer-overexpressed lncRNA by RNA sequencing, and it contributes to prostate cancer progression through regulation of target genes^21^. Further studies showed that PCAT1 increases cMYC and suppresses BRCA2 expression at the post-transcriptional level in prostate cancer^22,23^. Interestingly, PCAT1 is upregulated by a risk single nucleotide polymorphism (SNP) located in its enhancer region and interacts with AR and LSD1 to recruit them to the enhancers of GNMT and DHCR24 upon prolonged androgen treatment^24^. PCAT1 is also highly expressed and plays oncogenic roles in a variety of cancers, including gastric cancer^25^, lung cancer^26^ and hepatocellular carcinoma^27^, and it acts as a novel biomarker for poor prognosis^25,26,28^. Importantly, PCAT1 is reported to be upregulated in the serum of multiple myeloma patients and the urine of bladder cancer patients and is closely related to clinical diagnosis or prognosis^19,29^.

Elevated PCAT1 expression has also been observed in ESCC tissues and is associated with poor ESCC survival^30,31^. Preliminary studies showed that PCAT1 promotes the proliferation of ESCC cells^31,32^. However, the detailed biological functions, underlying mechanisms and clinical significance of PCAT1 in ESCC remain limited. In this study, we investigated the effects of PCAT1 overexpression and knockdown on the regulation of ESCC cell proliferation in vitro and in vivo. The interaction between PCAT1 and miR-326 was studied to reveal the underlying mechanisms of its action. We also determined whether PCAT1 expression is detectable in ESCC cell-derived exosomes and the serum of ESCC patients. Our study provides new insights into the molecular mechanisms and non-invasive biomarkers of ESCC.

## Results

### PCAT1 is highly expressed in human ESCC specimens and cell lines

**Fig. 1:**
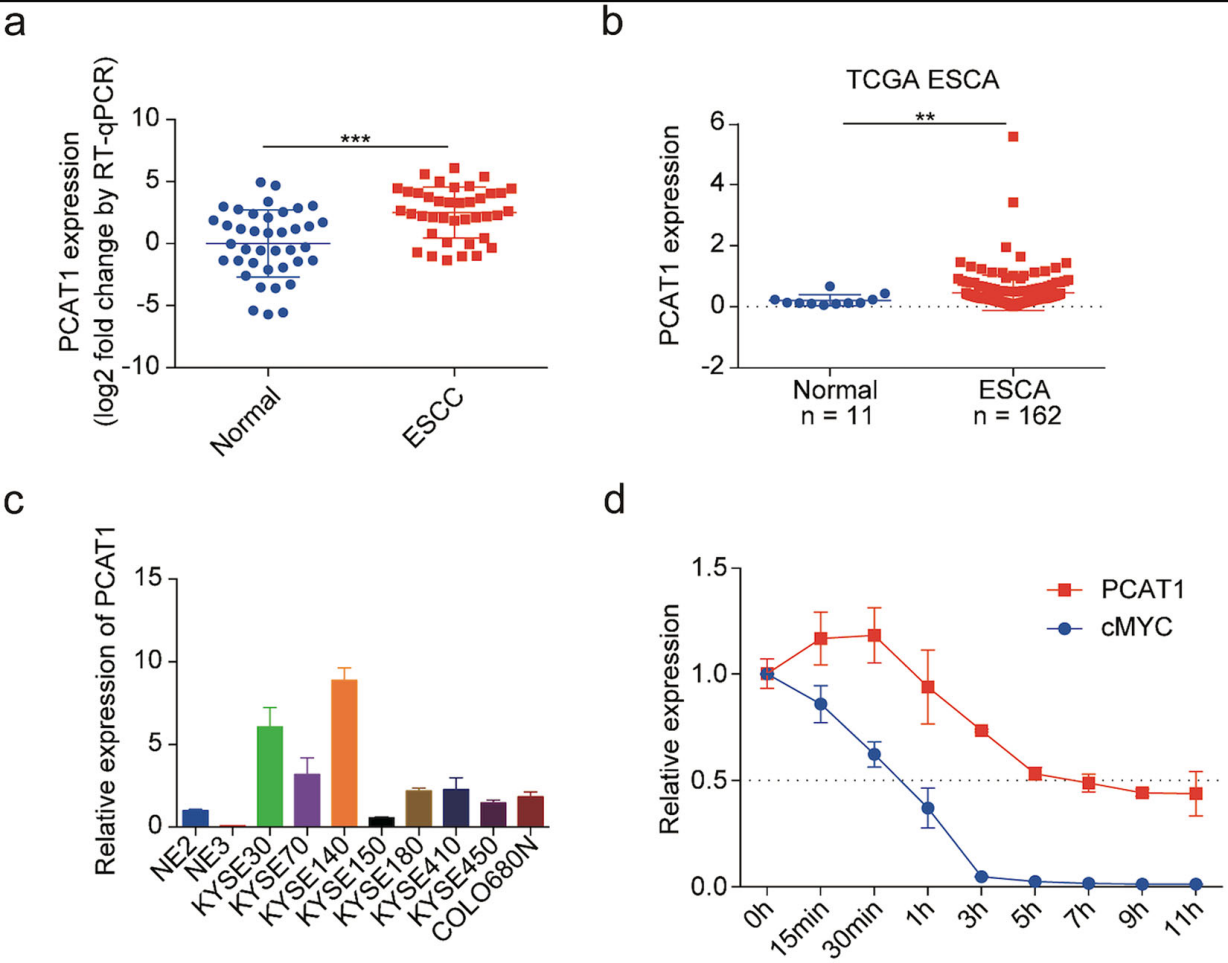
PCAT1 is upregulated in ESCC tissues and cell lines. **a** PCAT1 expression in 39 paired ESCC samples and adjacent normal tissues determined by RT-qPCR. β-actin was used as an internal reference. Statistical significance was assessed using the paired Student’s *t* test. ***P < 0.001. **b** Analysis of PCAT1 expression in ESCA (TCGA’s abbreviation for oesophageal carcinoma) tissues and normal tissues by using the TCGA database. Statistical significance was assessed using the Mann-Whitney test. **P < 0.01. **c** The expression of PCAT1 was detected in eight ESCC cell lines and two immortalized normal oesophageal epithelial cell lines (NE2 and NE3) by RT-qPCR. GAPDH was used as an internal reference. The expression of PCAT1 in NE2 cells was defined as 1. **d** RT-qPCR analysis of PCAT1 half-life in KYSE30 cells treated with actinomycin D. GAPDH was used as an internal reference

We evaluated the expression of PCAT1 in 39 paired human ESCC specimens and adjacent normal tissues and observed that PCAT1 was indeed highly expressed in ESCC specimens (Fig. 1a). Consistent results were obtained by analysis of the PCAT1 expression in oesophageal carcinoma using the Cancer Genome Atlas (TCGA) database^33^ (Fig. 1b). Next, we assessed PCAT1 expression in eight ESCC cell lines and two immortalized normal oesophageal epithelial cell lines. The results showed that PCAT1 expression was upregulated in ESCC cell lines compared with normal cells (Fig. 1c, S1). To detect the degradation of endogenous PCAT1, KYSE30 cells were harvested at the indicated times after treatment with 1 μg/mL actinomycin D. Total RNA was then isolated to detect the expression levels of PCAT1 and cMYC by RT-qPCR. We found that the half-life of PCAT1 was approximately 5 h, which is much longer than the half-life of cMYC (Fig. 1d). Collectively, these results indicate that PCAT1, a relatively stable lncRNA, is highly expressed in human ESCC specimens and cell lines.

### PCAT1 enhances the tumorigenicity of ESCC cells in vitro

**Fig. 2:**
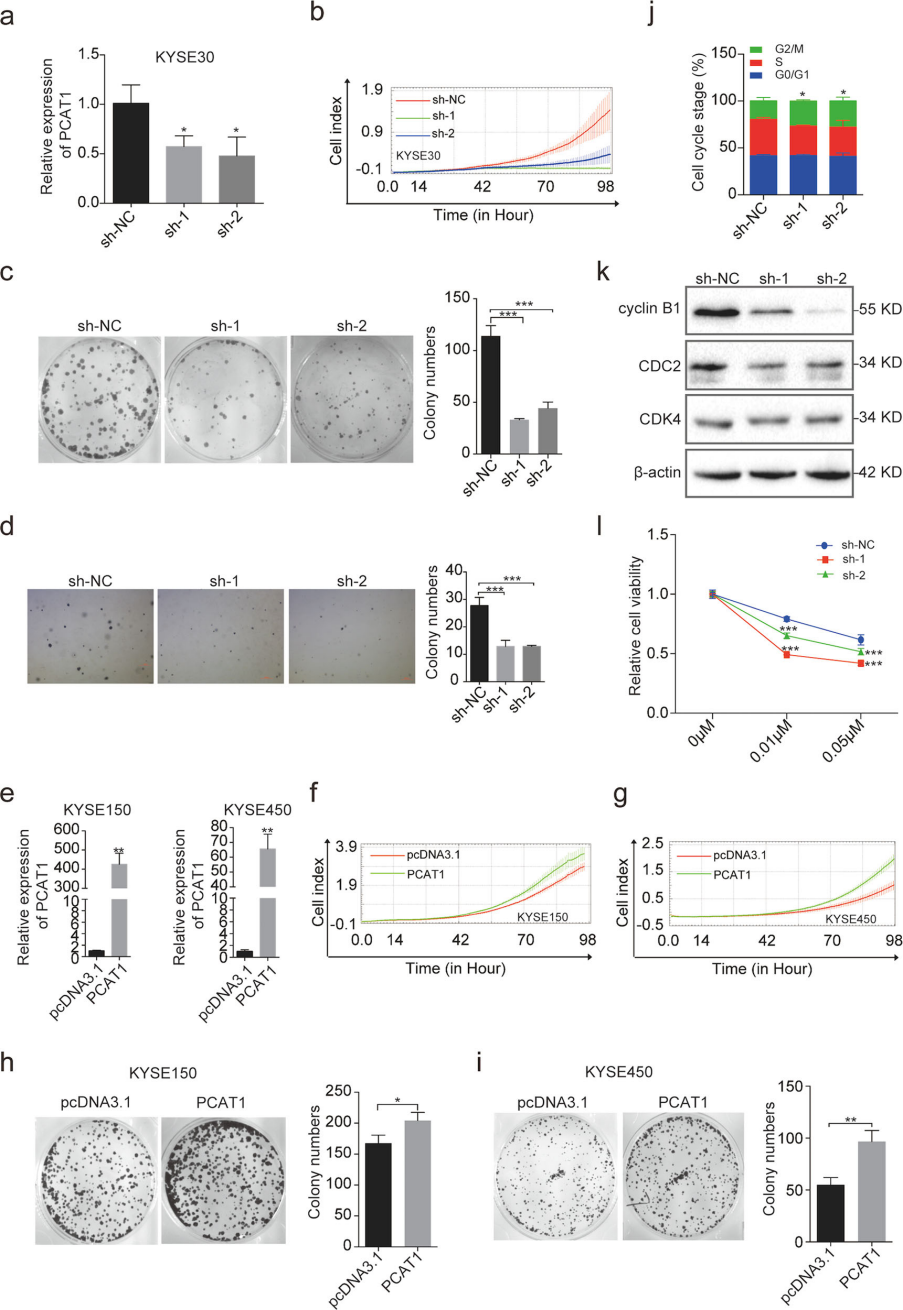
PCAT1 promotes ESCC cell proliferation, and knockdown of PCAT1 leads to G2/M phase arrest. **a** RT-qPCR analysis of PCAT1 expression in KYSE30 cells knocking down PCAT1. GAPDH was used as an internal reference. sh-NC: shRNA negative control, sh-1/2: shRNA targeting PCAT1. Statistical significance was assessed using one-way ANOVA. *P < 0.05 versus sh-NC. **b** The growth of KYSE30 cells with PCAT1 knockdown was monitored by the RTCA-MP system. **c** Colony formation assay was performed using KYSE30 cells with PCAT1 knockdown. Representative images (left) and quantification analysis (right) are shown (*n* = 3). Statistical significance was assessed using one-way ANOVA. ***P < 0.001 versus sh-NC. **d** A soft-agar colony formation assay was conducted using KYSE30 cells with PCAT1 knockdown. Representative images (left) and quantification analysis (right) are shown (*n* = 3). Statistical significance was assessed using one-way ANOVA. ***P < 0.001 versus sh-NC. **e** RT-qPCR analysis of PCAT1 expression in KYSE150 and KYSE450 cells overexpressing PCAT1. Statistical significance was assessed using two-tailed Student’s *t* test. **P < 0.01. **f**, **g** The growth of KYSE150 and KYSE450 cells with PCAT1 overexpression was monitored by the RTCA-MP system. **h**, **i** Colony formation assay was performed using KYSE150 and KYSE450 cells overexpressing PCAT1. Representative images (left) and quantification analysis (right) are shown (*n* = 3). Statistical significance was assessed using two-tailed Student’s *t* test. *P < 0.05; **P < 0.01. **j** The cell cycle distribution of KYSE30 cells with PCAT1 knockdown was determined by PI staining and flow cytometry. One-way ANOVA was used to compare the G2/M phase distribution (*n* = 3). *P < 0.05 versus sh-NC. **k** Western blot analysis of cyclin B1, CDC2 and CDK4 protein levels in KYSE30 cells with PCAT1 knockdown. **l** The paclitaxel sensitivity of KYSE30 cells with PCAT1 knockdown was determined with MTT. Statistical significance was assessed using two-way ANOVA. ***P < 0.001 versus sh-NC

To investigate the role of PCAT1 in ESCC tumorigenesis, we first stably knocked down PCAT1 expression using short hairpin RNA (shRNA) in KYSE30 cells (Fig. 2a), which express relatively high levels of PCAT1. Cell growth and colony formation assays were then utilized to evaluate the cell proliferation upon PCAT1 knockdown. The results showed that two shRNAs against PCAT1 (sh-1 and sh-2) reduced the growth rate and colony formation of KYSE30 cells compared to the negative control (sh-NC) (Fig. 2b, c). Furthermore, a soft-agar colony formation assay revealed that stable knockdown of PCAT1 significantly decreased soft-agar colony formation of KYSE30 cells (Fig. 2d), indicating that PCAT1 may play a role in maintaining oncogenesis of ESCC cell lines. Next, we selected KYSE150 and KYSE450 cells, which exhibit low levels of PCAT1, to stably express PCAT1. The successful overexpression of PCAT1 in the two cell lines was confirmed by RT-qPCR (Fig. 2e). As expected, the growth rate and colony formation of KYSE150 and KYSE450 cells with PCAT1 overexpression were increased compared with the negative control group (Fig. 2f–i). Taken together, these results suggest that PCAT1 promotes ESCC cell proliferation in vitro.

In addition, we analysed the cell cycle distribution in PCAT1-knockdown KYSE30 cells by flow cytometry. The results showed that stable knockdown of PCAT1 led to an increase in the percentage of cells in the G2/M phase (Fig. 2j). Consistent with that finding, PCAT1 downregulation decreased the expression of cyclin B1 and CDC2 (Fig. 2k). These results indicate that knockdown of PCAT1 causes G2/M arrest. Many anti-mitotic agents, such as paclitaxel, are effective in preventing the proliferation of tumour cells by targeting microtubules during M phase^34^. Thus, we speculated that PCAT1 may influence the response of ESCC cells to paclitaxel. Indeed, downregulation of PCAT1 resulted in an increase in cellular sensitivity to paclitaxel (Fig. 2l). These data strongly support a requirement of PCAT1 in ESCC cell growth and cell cycle progression.

### PCAT1 enhances the tumorigenicity of ESCC cells in vivo

**Fig. 3:**
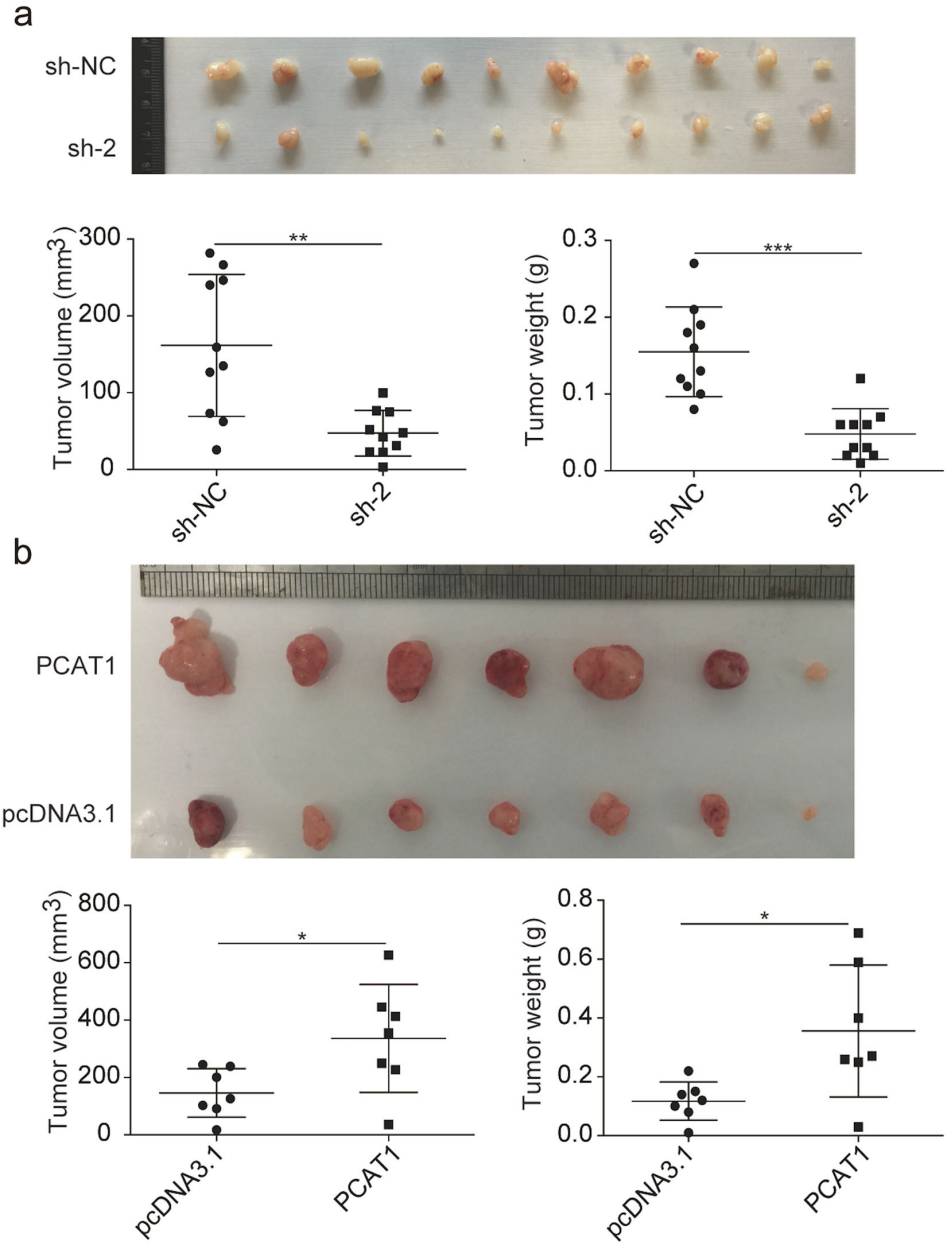
PCAT1 enhances ESCC cell growth in a nude mouse xenograft model. **a** Macroscopic appearance of the xenograft tumours in nude mice after injection with KYSE30-sh-NC and KYSE30-sh-2 cells for 4 weeks (upper, *n* = 10). Tumour weight and volume were examined (lower). Statistical significance was assessed using two-tailed Student’s *t* test. **P < 0.01; ***P < 0.001. **b** Macroscopic appearance of the xenograft tumours in nude mice after injection with KYSE450-pcDNA3.1 and KYSE450-PCAT1 cells for 4 weeks (upper, *n* = 7). Tumour weight and volume were examined (lower). Statistical significance was assessed using two-tailed Student’s *t* test. *P < 0.05

To further confirm the tumorigenic role of PCAT1 in ESCC, a tumour xenograft mouse model was made. KYSE30 control cells and PCAT1-knockdown cells, as well as KYSE450 control cells and PCAT1-overexpressed cells, were injected subcutaneously into nude mice. We subsequently observed that knockdown of PCAT1 led to smaller tumours compared with the KYSE30 control cells (Fig. 3a). The tumour weight of the PCAT1-knockdown group was also much lighter than that of the control group (Fig. 3a). On the other hand, injection of KYSE450 cells with higher PCAT1 expression led to larger and heavier tumours compared with control cells (Fig. 3b). Taken together, these results show that PCAT1 can significantly promote ESCC cell growth in vivo.

### PCAT1 functions as a ceRNA for miR-326

**Fig. 4:**
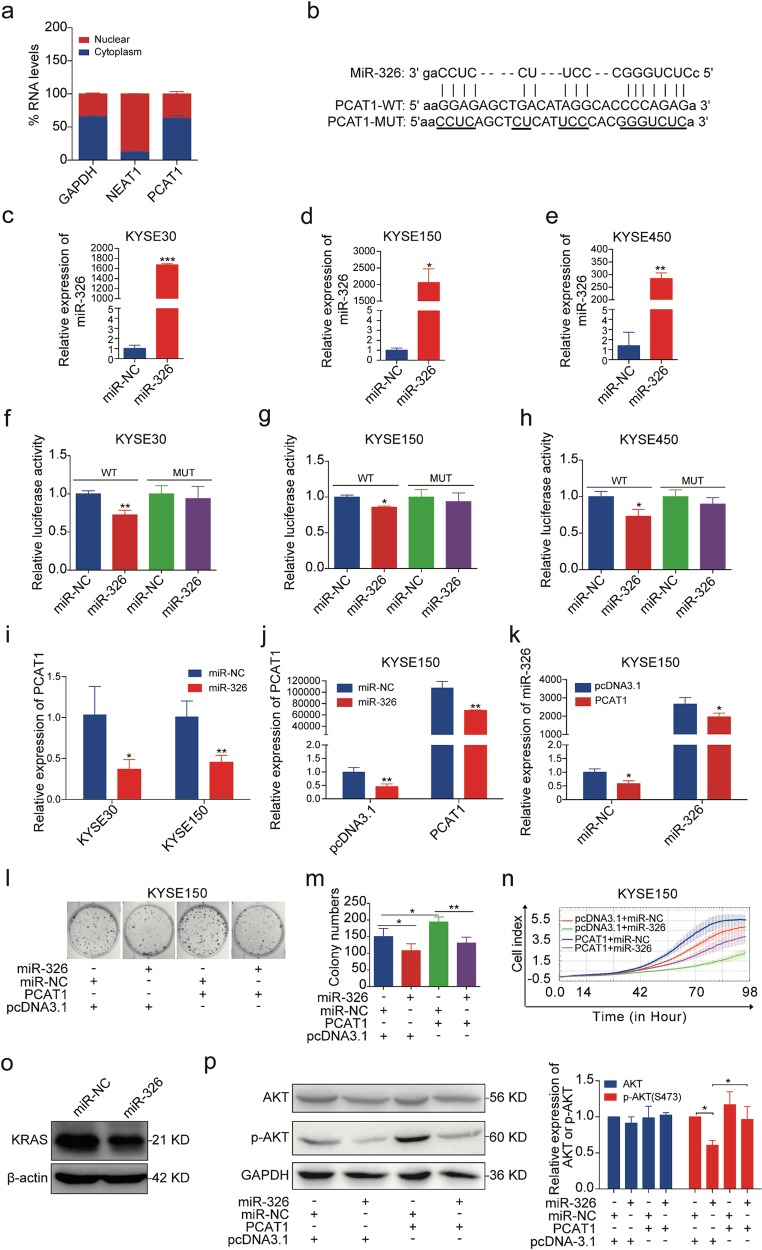
PCAT1 acts as a sponge of miR-326. **a** Fractionation of KYSE30 cells followed by RT-qPCR was used to determine the localization of PCAT1. GAPDH served as the control for cytoplasmic expression, and NEAT1 was the control for nuclear expression. **b** Schematic diagram of binding sites between PCAT1 and miR-326, as well as the mutation of binding sites in PCAT1. The mutated sequences are underlined. **c**–**e** RT-qPCR analysis of miR-326 expression in KYSE30, KYSE150 and KYSE450 cells after transfection of miR-326 mimics. U6 was used as an internal reference. Statistical significance was assessed using two-tailed Student’s *t* test. *P < 0.05; **P < 0.01; ***P < 0.001. **f**–**h** Dual-luciferase reporter assays showed the effect of miR-326 mimics on the luciferase activity of wild-type and mutant PCAT1 constructs in KYSE30, KYSE150 and KYSE450 cells. Statistical significance was assessed using two-tailed Student’s *t* test. *P < 0.05; **P < 0.01. **i** RT-qPCR analysis of PCAT1 expression in KYSE30 and KYSE150 cells after transfection of miR-326 mimics. GAPDH was used as an internal reference. Statistical significance was assessed using two-tailed Student’s *t* test. *P < 0.05; **P < 0.01. **j**, **k** RT-qPCR analysis of PCAT1 and miR-326 expression in KYSE150 cells co-transfected with PCAT1 vector and miR-326 mimics. GAPDH or U6 was used as an internal reference. Statistical significance was assessed using two-tailed Student’s *t* test. *P < 0.05; **P < 0.01. **l**, **m** Colony formation assay was performed in PCAT1-overexpressed KYSE150 cells transfected with miR-326 mimics. Representative images (**l**) and quantification analysis (**m**) are shown (*n* = 3). Statistical significance was assessed using one-way ANOVA. *P < 0.05; **P < 0.01. **n** The growth curve monitored by the RTCA-MP system was performed in PCAT1-overexpressed KYSE150 cells transfected with miR-326 mimics. **o** Western blot analysis of KRAS level in KYSE150 cells transfected with miR-326 mimics. **p** Western blot analysis of AKT and phospho-AKT (p-AKT) in PCAT1-overexpressed KYSE150 cells transfected with miR-326 mimics. Representative images (left) and quantification analysis (right) are shown (*n* = 3). Statistical significance was assessed using one-way ANOVA. *P < 0.05

We then aimed to investigate the mechanisms by which PCAT1 affects cell growth. To do this, the sub-cellular localization of PCAT1 was examined by RT-qPCR following cellular fractionation, and the results showed that most of the PCAT1 was localized in the cytoplasm of KYSE30 cells, while NEAT1 (a nuclear lncRNA) was confined to the nucleus (Fig. 4a). Emerging evidence suggests that cytoplasmic lncRNAs can act as competing endogenous RNAs (ceRNAs) to modulate the functions of microRNAs (miRNAs)^20,35^. To examine whether cytoplasm-localized PCAT1 can bind to endogenous miRNAs, we predicted its target miRNAs using RegRNA (http://regrna.mbc.nctu.edu.tw/html/prediction.html)^36^. According to the analysis, PCAT1 had recognition sites for several potential miRNAs, including miR-326 (Fig. 4b), which is a tumour suppressor in multiple human cancers^37–42^. Thus, miR-326 was selected for further study. A dual-luciferase reporter assay was performed to validate the interaction between them using a reporter plasmid with PCAT1 in the 3′UTR of the luciferase gene. Our data revealed that the luciferase activity of the wild-type PCAT1 reporter was suppressed by the transfection of the miR-326 mimics into KYSE30, KYSE150 or KYSE450 cells, while there was no significant change in the luciferase activity of the mutant PCAT1 reporter (Fig. 4f–h), suggesting that PCAT1 is a direct target of miR-326. The overexpression efficiency of the miR-326 mimics was confirmed by RT-qPCR (Fig. 4c–e). In accordance with the luciferase reporter assay, the introduction of the miR-326 mimics led to a decrease in the endogenous levels of PCAT1 in KYSE30 and KYSE150 cells (Fig. 4i). Similar results were observed after co-transfection of the miR-326 mimics with the PCAT1 vector (Fig. 4j). Interestingly, the expression of endogenous and exogenous miR-326 was also decreased when PCAT1 vector were co-transfected with the control mimics and miR-326 mimics, respectively (Fig. 4k). Furthermore, the promotion of proliferation and colony formation induced by PCAT1 overexpression was inhibited by the miR-326 mimics (Fig. 4l–n). Collectively, these results indicate that PCAT1 promotes ESCC cell growth by acting as a sponge of miR-326.

A recent study has reported that miR-326 serves as a tumour suppressor in melanoma by targeting KRAS and regulating the AKT and ERK signalling pathways^37^. We examined whether miR-326 could regulate AKT signalling pathway in ESCC cells. The results showed that the levels of KARS and p-AKT were reduced in miR-326-overexpressed KYSE150 cells (Fig. 4o, p), while the decreased p-AKT levels caused by miR-326 overexpression were markedly restored by co-transfection of PCAT1 (Fig. 4p). Our data demonstrate that PCAT1 functions as a ceRNA for miR-326 to protect AKT signalling pathway from miR-326-mediated inhibition.

### PCAT1 expression is elevated in the serum of ESCC patients

**Fig. 5:**
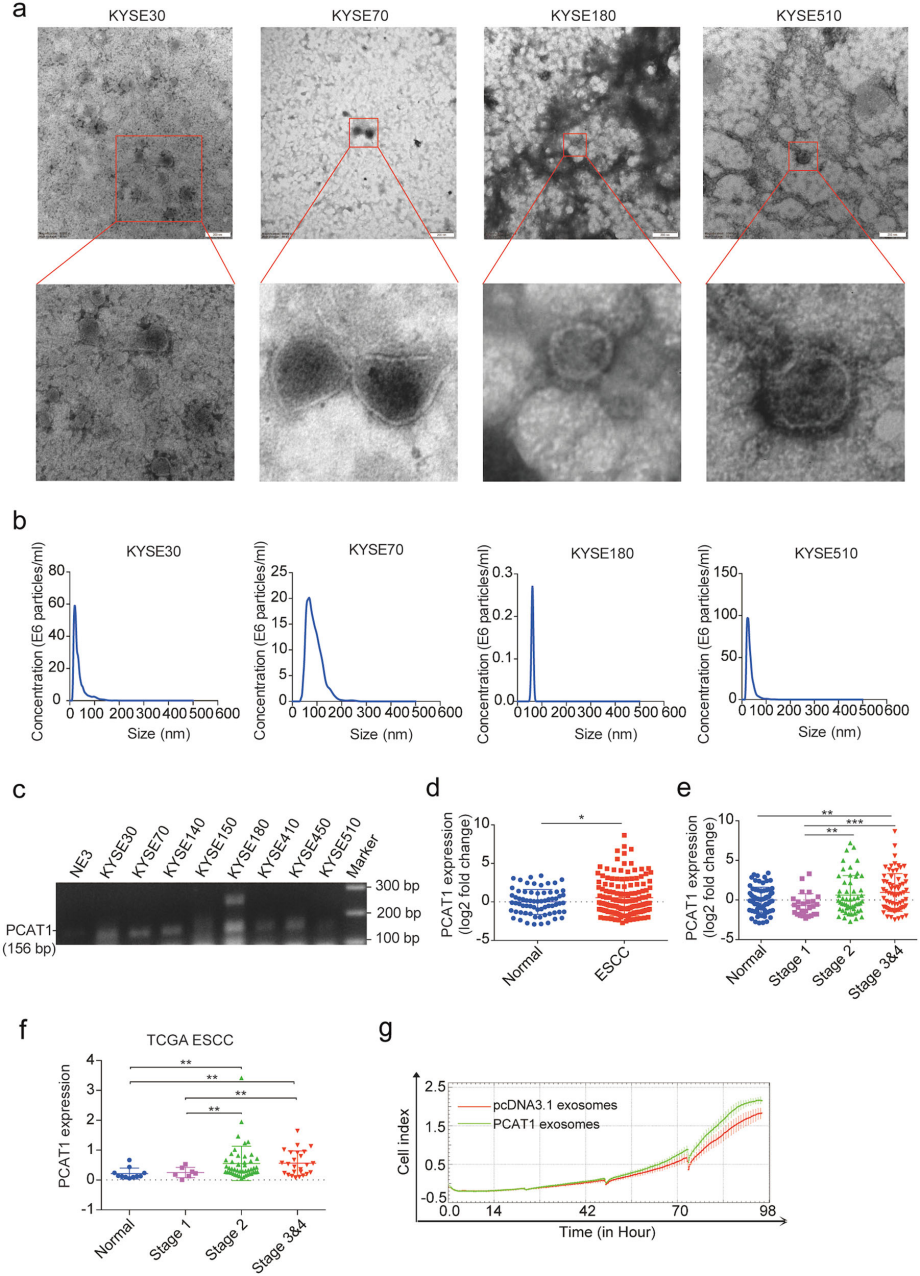
PCAT1 exists in ESCC cell-derived exosomes and is upregulated in the serum of ESCC patients. **a** Representative TEM images of exosomes secreted by KYSE30, KYSE70, KYSE180 and KYSE510 cells. Scale bar, 200 nm. **b** NTA analysis of the size distributions and numbers of exosomes derived from KYSE30, KYSE70, KYSE180 and KYSE510 cells. **c** RT-PCR analysis of PCAT1 expression in exosomes isolated from eight ESCC cell lines and one immortalized normal oesophageal epithelial cell line (NE3). **d** RT-qPCR analysis of PCAT1 expression in the serum of healthy donors (*n* = 69) and ESCC patients (*n* = 147). GADD7 was used as an exogenous reference. Statistical significance was assessed using two-tailed Student’s *t* test. *P < 0.05. **e** PCAT1 expression in the serum of healthy donors (*n* = 69) and ESCC patients with stage 1 (*n* = 26), stage 2 (*n* = 53) and stage 3 & 4 disease (*n* = 68). Statistical significance was assessed using two-tailed Student’s *t* test. **P < 0.01; ***P < 0.001. **f** Analysis of PCAT1 expression in ESCC tissues with stage 1 (*n* = 7), stage 2 (*n* = 47) and stage 3&4 (*n* = 25) and normal tissues (*n* = 11) using the TCGA database. Statistical significance was assessed using two-tailed Student’s *t* test. **P < 0.01. **g** The growth curve monitored by the RTCA-MP system was performed in NE3 cells with KYSE150-pcDNA3.1 and KYSE150-PCAT1-derived exosomes added at 0, 24, 48 and 72 h

Based on the overexpression and functional roles of PCAT1 in ESCC, we sought to determine whether PCAT1 can be secreted by ESCC cells through exosomes and serve as a non-invasive biomarker for ESCC. To explore this, exosomes were extracted from the cultured supernatant of ESCC cell lines by ultracentrifugation. The morphology of exosomes was verified as typical lipid bilayer membrane-encapsulated nanoparticles under a transmission electron microscope (TEM) (Fig. 5a). The size distribution and concentration of exosomes were analysed using nanoparticle tracking analysis (NTA). As shown in Fig. 5b, the isolated exosomes had a predominant size of 30–100 nm and a concentration ranging from 0.3 × 10^6^ to 100 × 10^6^ particles/ml. We then measured PCAT1 expression levels in ESCC cell-derived exosomes using RT-PCR. The results showed that PCAT1 was expressed highly in the ESCC cell-derived exosomes but weakly in those derived from immortalized normal oesophageal epithelial cells (Fig. 5c), indicating that PCAT1 can be secreted by ESCC cells.

We next investigated whether PCAT1 is present in the serum of ESCC patients. To explore circulating PCAT1, we isolated RNA from the serum of 147 ESCC patients and 69 healthy donors and assessed the expression of PCAT1 by RT-qPCR. Indeed, the expression of PCAT1 was detectable in the serum of ESCC patients and was significantly upregulated compared with healthy controls (Fig. 5d). Moreover, the levels of PCAT1 were higher in the serum from advanced ESCC patients than those from stage 1 ESCC patients (Fig. 5e). We then analysed the expression of PCAT1 in different stages of ESCC tissues from the TCGA database and found elevated levels of PCAT1 in advanced tumours (Fig. 5f), which is consistent with its increase in the serum of patients with advanced tumours.

To study the role of exosomes from PCAT1-overexpressed cells in cell growth, equivalent numbers of KYSE150-pcDNA3.1 and KYSE150-PCAT1 cells were plated in exosome-free medium. After the cells were cultured for 48 h, exosomes were isolated from the supernatant and added to the immortalized normal oesophageal epithelial cell line NE3 at 0, 24, 48 and 72 h. We observed that exosomes derived from KYSE150-PCAT1 cells obviously increased NE3 cell proliferation when compared with those derived from KYSE150-pcDNA3.1 cells (Fig. 5g). Taken together, these results demonstrate that PCAT1 is present in the exosomes of ESCC cells and the serum of ESCC patients and promotes the cell growth through exosomes.

## Discussion

Oesophageal cancer ranks as one of the most prevalent cancers and the fourth leading cause of cancer death in China^43^. To improve the survival rate of ESCC patients, a better understanding of the underlying mechanisms and a search for potential biomarkers are urgently required. Emerging evidence has indicated that lncRNAs play critical roles in the progression of ESCC^44,45^. In the present study, we found that PCAT1 was overexpressed in ESCC tissues and cell lines and promoted ESCC cell proliferation by sponging miR-326. Importantly, PCAT1 was packaged into ESCC cell-derived exosomes and highly expressed in the serum of ESCC patients compared with healthy controls.

To confirm the high levels of PCAT1 in ESCC, we examined the expression of PCAT1 in ESCC tissues and cell lines and obtained results consistent with previous reports^30,31^. Knockdown of PCAT1 has been shown to inhibit malignant phenotypes in several human cancers, such as gastric cancer^25^, lung cancer^26^ and hepatocellular carcinoma^27^. Here, we found that knockdown of PCAT1 decreased the growth and colony formation of KYSE30 cells, while overexpression of PCAT1 increased the growth and colony formation of KYSE150 and KYSE450 cells. Consistent with these results was the growth of xenograft tumours upon either overexpression or knockdown of PCAT1 in vivo. Furthermore, we observed that knockdown of PCAT1 induced cell cycle arrest in G2/M phase and enhanced the cellular response to paclitaxel. A recent study showed that depletion of PCAT1 inhibits the growth of oesophageal cancer and enhances its chemosensitivity to cisplatin^32^, which further supports our conclusions. Collectively, these findings demonstrate that PCAT1 may have an oncogenic role in ESCC.

Studies have reported that miR-326 is downregulated and functions as a tumour suppressor in some types of human cancers, including melanoma^37^, gastric cancer^38^, non-small-cell lung cancer^39^, osteosarcoma^40,41^ and gliomas^42^. LncRNAs can regulate miRNA activity through base-pairing interactions, and such lncRNAs are a category of transcripts termed ceRNAs^46^. For instance, lncARSR enhances the expression of AXL and c-MET by competitively binding miR-34/miR-449 and thereby promotes RCC cell sunitinib resistance^20^. LncRNA-ATB induces EMT and invasion by competitively binding miR-200 family members to upregulate ZEB1 and ZEB2 expression^35^. PCAT1 can also function as a ceRNA to reverse HMGB1 expression by sponging miR-129-5p in hepatocellular carcinoma^47^ and is targeted by miR-145-5p in prostate cancer^48^. Here, miR-326 was predicted to target PCAT1, and a dual-luciferase reporter assay demonstrated that there was indeed a functional interaction between miR-326 and PCAT1 in ESCC cells. Furthermore, the miR-326 mimics led to a decrease in PCAT1 expression levels. Rescue experiments revealed that miR-326 overexpression attenuated the promotive effect of PCAT1 on ESCC cell growth. Thus, to the best of our knowledge, our data are the first to show that PCAT1 promotes ESCC cell growth by sponging miR-326.

LncRNAs not only are involved in carcinogenesis and serve as promising therapeutic targets but also may be novel non-invasive biomarkers detectable in body fluids, especially blood^49^. In 2015, Li’s group^50^ found that circulating HULC and LINC00152 were significantly upregulated in HCC patient plasma. Tong et al.^51^ measured 10 ESCC-related lncRNAs in plasma from ESCC patients and healthy volunteer donors and found that the plasma levels of POU3F3, HNF1A-AS1 and SPRY4-IT1 were much higher in ESCC patients. Our current study showed that PCAT1 could be detected in exosomes derived from ESCC cells and was upregulated in serum of ESCC patients compared with healthy volunteer donors, which is consistent with the high levels of PCAT1 in the serum of multiple myeloma patients^29^ and the urine of bladder cancer patients^19^. With the combination of its relative stability and its overexpression in ESCC tissues, PCAT1 might serve as a potential biomarker for the diagnosis of ESCC. Moreover, circulating PCAT1 may promote tumour proliferation or chemoresistance to paclitaxel via exosomes transmitted to close or distant cells. In our research, exosomes derived from KYSE150-PCAT1 cells increased NE3 cell proliferation compared to those derived from KYSE150-pcDNA3.1 cells, indicating that PCAT1 promotes cell growth through exosomes. Recently, many studies have demonstrated that miRNAs and lncRNAs can be packaged into exosomes and affect the chemoresistance, proliferation, metastasis, apoptosis and energy metabolism of tumour cells in a variety of ways^20,52–54^.

In summary, we further identified PCAT1 as an oncogene in ESCC and describe a novel mechanism in which PCAT1 binds to miR-326 to promote ESCC progression. Moreover, PCAT1 is highly expressed in the serum of ESCC patients. Our findings demonstrate that PCAT1 may act as not only a therapeutic target but also a non-invasive potential biomarker for ESCC patients.

## Materials and methods

### Tissue specimens and cell culture

Thirty-nine paired ESCC samples and adjacent normal tissues of patients with ESCC were collected from the Fourth Hospital of Hebei Medical University. This study was approved by the Ethics Committee of the Fourth Hospital of Hebei Medical University, and informed consent was obtained. The serum samples of ESCC patients were obtained from Peking University Cancer Hospital Biobank, while the serum samples of healthy donors were collected from the Cancer Hospital, Chinese Academy of Medical Sciences and Peking Union Medical College. Research was approved by the corresponding Ethics Committees, and informed consent was obtained.

ESCC cell lines (KYSE30, KYSE70, KYSE140, KYSE150, KYSE180, KYSE410, KYSE450, KYSE510 and COLO680N), kindly provided by Professor Yutaka Shimada of Kyoto University, were cultured in RPMI 1640 (Gibco) with 10% foetal bovine serum (FBS). Immortalized normal oesophageal epithelium cell lines NE2 and NE3 were cultured in a 1:1 mixture of EpiLife and dKSFM (Gibco). All of these cells were maintained at 37 °C in a humidified atmosphere containing 5% CO_2_.

### RNA extraction, reverse transcription-quantitative real-time PCR (RT-qPCR) and semi-quantitative PCR

Total RNA was isolated using Trizol reagent (Invitrogen, USA). First-strand cDNA was generated using the PrimeScript^TM^II 1st Strand cDNA Synthesis Kit (Takara, China). Real-time PCR was performed in the QuantStudio Real-Time PCR System (Applied Biosystems, USA) and the Mx3000P Real-Time PCR System (Agilent, USA) using SYBR Premix Ex Taq™ II (Takara, China), and the gene-specific primers are shown in Supplementary Table 1. GAPDH or β-actin was employed as an endogenous control for mRNA and lncRNA. For microRNA analysis, real-time PCR was performed as above, and U6 was employed as an endogenous control. The PCAT1 levels in serum were normalized against a synthesized exogenous reference, GADD7, using a MEGAscript® Kit (Life Technologies, USA). The relative expression of RNAs was calculated using the 2^–ΔΔCt^ method.

Semi-qualitative PCR was conducted with 2 × Taq PCR Master Mix (TIANGEN, China) on a Veriti 96-well Thermal Cycler (Applied Biosystems, USA) using the following conditions: 95 °C for 5 min; then 40 cycles of (95 °C for 30 s, 60 °C for 30 s, and 72 °C for 30 s); and finally 72 °C for 5 min. The amplified products were analysed on a 1.5% agarose gel.

### Determination of PCAT1 half-life

KYSE30 cells grown to <70% confluency were treated with actinomycin D (1 µg/mL, Sigma-Aldrich, USA) to inhibit transcription. Cells were then collected in Trizol reagent at the indicated time points.

### Isolation of cytoplasmic and nuclear fractions

The KYSE30 cells were harvested and washed with phosphate-buffered saline (PBS), and then centrifuged at 500 × *g* for 5 min. After removal of the PBS, the cell pellet was resuspended with 100 μL of nuclear and cytoplasmic extraction reagent (140 mM NaCl, 1.5 mM MgCl_2_, 10 mM Tris-HCl pH 8.5, 0.5% NP-40), incubated on ice for 5 min, and centrifuged at 5000 × *g* for another 5 min. The supernatant was then removed to a new tube, and 1 mL Trizol reagent was added. After washing with PBS, the nuclear pellet was also resuspended in 1 mL of Trizol reagent. RNA extracted from each of the fractions was subjected to RT-qPCR analysis to detect the levels of nuclear control transcript (NEAT1), cytoplasmic control transcript (GAPDH) and PCAT1.

### Vector constructions

To obtain the pcDNA3.1-PCAT1 vector, the cDNA of PCAT1 was PCR-amplified by the PrimeSTAR® HS DNA Polymerase (Takara, China) and cloned into the *Hind* III and *Xba*I sites of the pcDNA3.1 vector. PCAT1 shRNA-1 (sh-1) and PCAT1 shRNA-2 (sh-2) were synthesized by GenePharma (Shanghai, China) and cloned into the pGPH1/Neo vector. The pmirGLO plasmid with PCAT1 in the 3′UTR of the luciferase gene (pmirGLO-PCAT1-WT) was constructed by Generay (Shanghai, China). The pmirGLO-PCAT1-MUT with point mutations in miR-326 binding sites was mutated from pmirGLO-PCAT1-WT by Generay (Shanghai, China). PCAT1 full-length primers used for amplification of PCAT1 and shRNA sequences targeting PCAT1 are shown in Supplementary Table 2.

### Establishment of cell lines stably expressing or knocking down PCAT1

To obtain cell lines stably expressing PCAT1, KYSE150 and KYSE450 cells were transfected with the pcDNA3.1 control plasmid or pcDNA3.1-PCAT1 plasmid using Lipofectamine 2000 (Invitrogen, USA) according to the manufacturer’s instructions and then selected with G418 (300 μg/mL) for 2 weeks. To generate cell lines with stable knockdown of PCAT1, KYSE30 cells were transfected with the pGPH1/Neo control plasmid (sh-NC) or PCAT1-sh-1/sh-2 plasmid using Lipofectamine 2000 and selected with G418 (300 μg/mL) for 2 weeks. The cell lines with stable overexpression or knockdown of PCAT1 were identified using RT-qPCR.

### Cell growth and drug sensitive assay

The growth ability of ESCC cells was evaluated using the xCELLigence Real-Time Cell Analyzer (RTCA)-MP system (Acea Biosciences/Roche Applied Science), which can detect the cellular growth status in real time. First, 100 μL of RPMI 1640 complete medium was added to each well of an E-Plate 96 (Roche Applied Science) to obtain equilibrium. Then, 2 × 10^3^ cells in 100 μL of RPMI 1640 complete medium were seeded in each well. The E-Plate 96 was locked in the RTCA-MP device and continually cultured at 37 °C with 5% CO_2_. The cell index obtained from changes in electrical impedance directly reflects cellular proliferation on biocompatible microelectrode-coated surfaces and was read automatically every 15 min until the end of the experiment.

For the drug-sensitive assay, briefly, 5 × 10^3^ cells per well were plated in 96-well plates. After overnight culture, the cells were treated with a series of concentrations of paclitaxel. 48 h later, 10 μL of MTT solution was added to each well, followed by incubation for 2 h and subsequent addition of DMSO. The plate was then shaken and measured with a microplate reader (Thermo Scientific, USA) using a 492 nm filter.

### Colony formation assay

1 × 10^3^ cells per well were seeded into 6-well plates. After culture for 10 days, each well was washed three times with PBS, fixed with methyl alcohol for 10 min, and then stained with 0.5% crystal violet solution for 10 min. After washing with water, the colonies in each group were imaged and counted.

### Soft-agar colony formation assay

2 mL of 0.6% agarose gel with RPMI 1640 complete medium was placed into 6-well plates. Then, cells (1 × 10^4^ per well) in 0.4% agarose with RPMI 1640 complete medium were plated on top of a solidified layer, and 200 μL of RPMI 1640 complete medium was added every two days. These cells were continuously cultured for 30 days at 37 °C with 5% CO_2_. The colonies in each group were imaged and counted.

### Cell cycle analysis

The cells were harvested and washed with PBS, and then fixed with 70% ethanol overnight at −20 °C. The fixed cells were washed with PBS again and stained with 500 μL of propidium iodide (PI) buffer (50 μg/mL PI, 50 μg/mL RNaseA, 0.1% Triton) for 30 min at 37 °C followed by analysis using flow cytometry (BD Biosciences). The percentages of cells in different phases were compared.

### Transient transfection

Transfection was performed using the Lipofectamine 2000 reagent (Invitrogen, USA) according to the manufacturer’s instructions. The double-stranded miR-326 mimics and negative control RNAs (miR-NC) (RiboBio, China) were introduced into cells that were harvested 48 h after transfection.

### Luciferase reporter assay

The pmirGLO-PCAT1-WT or pmirGLO-PCAT1-MUT vector was cotransfected with miR-326 mimics or miR-NC into KYSE30, KYSE150 and KYSE450 cells using Lipofectamine 2000 (Invitrogen, USA) according to the manufacturer’s protocols. After 24 h of transfection, the firefly and Renilla luciferase activities were assessed by the Dual-Luciferase Reporter Assay System (Promega, USA). The relative firefly luciferase activity was normalized to Renilla luciferase activity.

### Western blot analysis

Total cellular lysates were prepared in RIPA lysis buffer (APPLYGEN, China). Identical quantities of proteins were separated by sodium dodecyl sulfate-polyacrylamide gel electrophoresis (SDS-PAGE) and transferred onto PVDF membranes. After incubation with antibodies specific for cyclin B1 (Proteintech, USA), CDC2 (Santa Cruz Biotechnology, USA), CDK4 (Proteintech, USA), KRAS (Proteintech, USA), AKT (CST, USA), p-AKT (CST, USA), GAPDH (Proteintech, USA) or β-actin (Sigma-Aldrich, USA), the membranes were incubated with horseradish peroxidase (HRP)-conjugated anti-rabbit IgG or anti-mouse IgG secondary antibody, and bands were detected using the ImageQuant™ LAS 4000 (GE Healthcare Life Sciences, USA) and the ChemiDoc^TM^ XRS + (Bio-Rad, USA). β-actin or GAPDH was used as a loading control.

### Animal studies

KYSE30-sh-NC and KYSE30-sh-2 cells (1 × 10^6^) were injected into the left backside region and the right backside region of 6-week-old male athymic nude mice, respectively (*n* = 10/group). KYSE450-pcDNA3.1 and KYSE450-PCAT1 cells (1 × 10^6^) were injected into the left backside region and the right backside region, respectively (*n* = 7/group). Tumour size was measured using calliper. Tumour volume was calculated using the formula (L × W^2^)/2, where L is the length and W is the width of the tumour. All animal experiments were approved by the Institutional Animal Care and Use Committee of National Cancer Center/National Clinical Research Center for Cancer/Cancer Hospital, Chinese Academy of Medical Sciences and Peking Union Medical College.

### Exosome experiments

Exosomes were collected through standard centrifugation steps as previously described^55^. FBS was ultracentrifuged at 120,000 × *g* for 70 min to remove exosomes. ESCC cells were cultured in exosome-depleted medium. The supernatant was collected after incubation for 48–60 h and centrifuged at 3000 rpm for 20 min. Then, the supernatant was centrifuged again at 12,000 × *g* for 45 min to remove the cell debris and large EVs. After filtration through a 0.22 μm Millex-GV filter unit (Millipore), the supernatant was centrifuged at 120,000 × *g* for 70 min. After removal of the supernatant, the precipitate was retained, washed with PBS, and centrifuged at 120,000 × *g* for 70 min. The precipitate was then dissolved in PBS and stored at 4 °C. Exosomes were examined by transmission electron microscope (TEM-1400 Plus) using negative staining and quantified by a NanoSight NS300 instrument (Malvern Instruments Ltd. UK) equipped with NTA 3.0 analytical software (Malvern Instruments Ltd. UK).

### In vitro exosome transfer

Exosome collection: Equivalent numbers of KYSE150-pcDNA3.1 and KYSE150-PCAT1 cells were plated in exosome-free medium and cultured for 48 h. The supernatant from cells was collected and then centrifuged at 3000 rpm for 20 min to remove cell debris. The supernatant was concentrated to 1 mL using a Millipore ultrafiltration centrifuge tube (Millipore, USA).

Exosome isolation: Exosomes in supernatant were isolated with the Ribo^TM^ Exosome Isolation Reagent (RiboBio, China) according to the following protocols: The concentrated supernatant was transferred to a new tube, and 1/3 volume of Ribo^TM^ Exosome Isolation Reagent was added. Each sample was mixed well by inverting or flicking the tube, and it was incubated at 4 °C overnight, after which the solution appeared cloudy. Each sample was centrifuged at 15,000 × *g* for 2 min at 4 °C. We carefully aspirated off the supernatant, and resuspended the pellet in 400 μL of PBS, and stored it at 4 °C.

Exosome treatment: NE3 cells were seeded at 2 × 10^3^ cells/well into an E-Plate 96 (Roche Applied Science), and 5 μL of KYSE150-pcDNA3.1 and KYSE150-pcDNA-PCAT1-derived exosomes were added at 0, 24, 48 and 72 h. Cell growth was monitored by the RTCA-MP system at 37 °C with 5% CO_2_.

### Statistical analysis

Data are presented as the mean ± standard deviation (SD). Comparisons were determined using unpaired/paired Student’s *t* test, Mann-Whitney test, one-way ANOVA or two-way ANOVA (*P < 0.05, **P < 0.01 and ***P < 0.001) as indicated in individual figures. The differences were deemed statistically significant at *P* < 0.05.

## Supplementary information

Tables

Figure S1
