# Supplementary material for: Long noncoding RNA PCAT1, a novel serum-based biomarker, enhances cell growth by sponging miR-326 in oesophageal squamous cell carcinoma
Source: Cell Death Dis. 2019 Jul 4;10(7):513. doi: 10.1038/s41419-019-1745-4 (PMC6609620; doi:10.1038/s41419-019-1745-4)
Supplement: Supplementary file 1 — Tables [file 41419_2019_1745_MOESM1_ESM.docx]

| Gene | Sequence of the primers |
| --- | --- |
| PCAT1 sense | 5'-TGAGAAGAGAAATCTATTGGAACC-3' |
| PCAT1 anti-sense | 5'-GGTTTGTCTCCGCTGCTTTA-3' |
| GAPDH sense | 5'-ATGGGGAAGGTGAAGGTCG-3' |
| GAPDH anti-sense | 5'- GGGGTCATTGATGGCAACAATA-3' |
| β-actin sense | 5'- GGGAAATCGTGCGTGACATTAAG-3' |
| β-actin anti-sense | 5'- TGTGTTGGCGTACAGGTCTTTG-3' |
| U6 sense | 5'-GCTTCGGCAGCACATATACTAAAAT-3' |
| U6 anti-sense | 5'-CGCTTCACGAATTTGCGTGTCAT-3' |
| NEAT1 sense | 5'-AGTTAAGGCGCCATCCTCAC-3' |
| NEAT1 anti-sense | 5'-AGCACTGCCACCTGGAAAAT-3' |
| miR-326  reverse transcription | 5'GTCGTATCCAGTGCAGGGTCCGAGGTATT  CGCACTGGATACGACCTGGAG-3' |
| miR-326 sense | 5'-GAGTGCCTCTGGGCCCTTC-3' |
| miR-326 anti-sense | 5'-GTGCAGGGTCCGAGGT-3' |

Supplementary Table 1. Primer sequences used for qPCR assays

Supplementary Table 2. Primer sequences used for vectors construction

| Gene | Sequence of the primers |
| --- | --- |
| PCAT1 full length-F | 5'-CCCAAGCTTACACATGGATATTGGA  TATCTGCAT-3' |
| PCAT1 full length-R  PCAT1 shRNA-1  PCAT1 shRNA-2 | 5'-TGCTCTAGATAGGCTCAAACACACATTT  ATTCATC-3'  5'-GCAGAAACACCAAUGGAUAUU-3'  5'-AUACAUAAGACCAUGGAAAU-3' |
