# Supplementary figures and images for: Long noncoding RNA PCAT1, a novel serum-based biomarker, enhances cell growth by sponging miR-326 in oesophageal squamous cell carcinoma
Source: Cell Death Dis. 2019 Jul 4;10(7):513. doi: 10.1038/s41419-019-1745-4 (PMC6609620; doi:10.1038/s41419-019-1745-4)

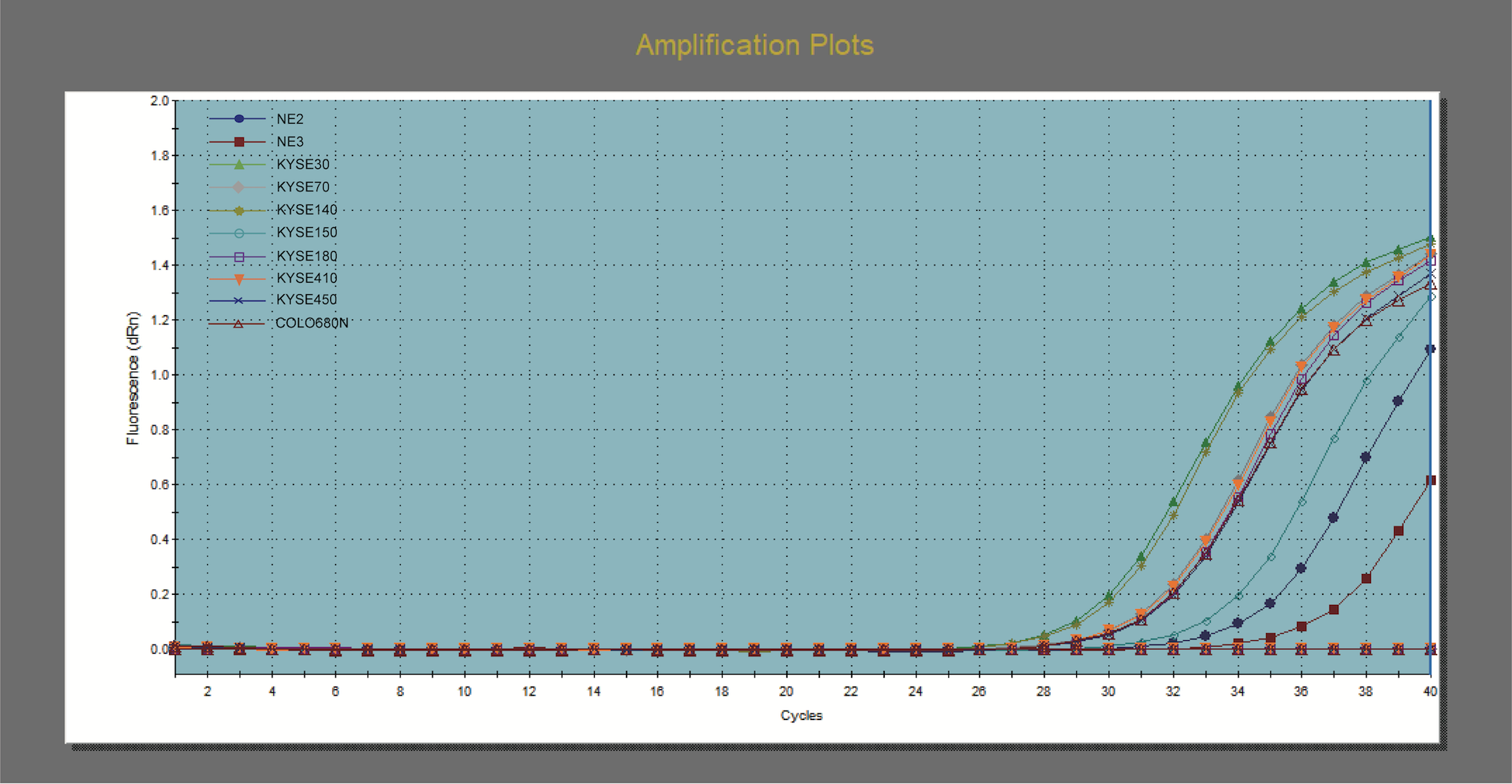

Supplement: Supplementary file 2 — Figure S1 [file 41419_2019_1745_MOESM2_ESM.tif]
